# Temporal trends in reported vector-borne diseases in China before and after the onset of COVID-19 from 2004 to 2023

**DOI:** 10.1371/journal.pntd.0014745

**Published:** 2026-10-21

**Authors:** Zhiqun Lei, Qianying Liu, Zhixin Lei

**Affiliations:** 1 School of Nursing and Health Management, Institute of Biomedicine, Wuhan Donghu University, Wuhan, Hubei, China; 2 School of NextMed Science and Technology, Hubei Key Laboratory of Nanomedicine for Neurodegenerative Disease, Institute of WUT-AMU, Wuhan University of Technology, Wuhan, China; 3 Hubei Key Laboratory of Gynecologic Oncology and Reproductive Health, Hubei Provincial Clinical Research Center for Cervical Lesions, Xiangyang, Hubei, China; Oregon State University College of Veterinary Medicine, UNITED STATES OF AMERICA

## Abstract

**Background:**

Vector-borne diseases (VBDs) continue to pose a public health burden in China. However, changes in the reported incidence and mortality of VBDs during the COVID-19 period remain insufficiently characterized.

**Methods:**

We analyzed five major VBDs—typhus, dengue, leishmaniasis, malaria, and epidemic encephalitis B (EEB)—in China from 2004 to 2023. Province- and age-specific surveillance data for 2004–2020 were obtained from the Chinese Center for Disease Control and Prevention, whereas national aggregate data for 2021–2023 were obtained from the National Health Commission. Accordingly, spatial and age-specific analyses were restricted to 2004–2020, while national trend analyses were extended through 2023. Annual trends were evaluated using Joinpoint regression, monthly seasonal patterns were assessed using STL decomposition, monthly changes after January 2020 were evaluated using interrupted time-series analysis, and expected monthly trajectories for 2020–2023 were estimated using SARIMA models trained on pre-COVID data.

**Results:**

From 2004 to 2020, incidence and mortality of VBDs declined significantly, especially for malaria, dengue, and EEB, and spatial hotspots were concentrated mainly in southern provinces. Compared with 2013–2019, overall incidence and mortality during 2020–2023 were 59.86% lower (95% CI: –60.36 to –59.36; P < 0.001) and 83.72% lower (95% CI: –87.86 to –78.18; P < 0.001), respectively. Malaria and EEB showed the largest reductions, whereas typhus and leishmaniasis changed less markedly. However, these declines should be interpreted cautiously because they may reflect a combination of COVID-19-era public health measures, pre-existing long-term trends, disease-specific control programs, and possible surveillance disruption.

**Conclusions:**

During the COVID-19 period in China, reported VBD incidence and mortality decreased substantially. However, it would be overly simplistic to attribute these changes entirely to COVID-19-related interventions, as other factors were also likely involved. These findings highlight the need for reliable surveillance and reporting during public health emergencies, together with forecasting tools that can be adapted to changing conditions, to support future vector-borne disease control.

## Author summary

Vector-borne diseases, such as dengue, malaria, typhus, leishmaniasis, and Japanese encephalitis, remain important public health problems because their spread is shaped by insects, human movement, climate, and health services. In this study, we examined how these diseases changed in China over two decades, with particular attention to the years affected by COVID-19. We found that reported cases and deaths decreased substantially over time, and that several diseases were reported less often during 2020–2023 than would have been expected from earlier patterns. The largest decreases were seen for malaria and Japanese encephalitis, while typhus and leishmaniasis changed less clearly. We also found that disease patterns differed by region and age group, with higher burdens in several southern and western areas. These findings are important because they show that large public health emergencies can affect the reporting and apparent burden of diseases beyond the emergency itself. We caution that the observed declines may reflect several factors, including long-term control programs, changes in travel and behavior, and possible disruption of routine surveillance. Reliable disease monitoring remains essential during future emergencies.

## Introduction

Vector-borne diseases (VBDs) are infections spread by arthropods such as mosquitoes, ticks, and sandflies, and they continue to place a substantial burden on public health worldwide [1]. The World Health Organization (WHO) estimates that VBDs account for more than 17% of all infectious diseases and are responsible for over 700,000 deaths each year [2]. Diseases such as dengue, malaria, leishmaniasis, and encephalitis remain particularly important because of their wide distribution and heavy disease burden, especially in tropical and subtropical regions.

Due to their strong associations with environmental, climatic, and socioeconomic factors, VBDs often display heterogeneous spatial and temporal patterns. Climate change, urbanization, and international travel have further complicated vector ecology and expanded risk areas [3,4]. Therefore, continuous surveillance and spatiotemporal monitoring are critical for early detection, trend forecasting, and effective public health responses [5,6]. China provides a particularly relevant setting for such work because of its large territory, diverse climate zones, and complex ecological environments [7]. Several VBDs are reported regularly in China, including typhus, dengue fever, leishmaniasis, malaria, and epidemic encephalitis B (EEB) [8,9]. Although China was certified malaria-free in 2021, the continued presence of competent vectors and the occurrence of imported cases mean that the risk has not disappeared entirely [10–12].

Over the past few decades, China has made substantial progress in controlling VBDs through vector control programs, improvements in sanitation, and better access to healthcare [13]. Even so, VBD prevention remains challenging. Dengue outbreaks continue to occur in some southern provinces, and leishmaniasis has reappeared or been reported in areas where it had previously been uncommon [14]. These patterns suggest that sustained vigilance is still needed. The COVID-19 pandemic added another layer of complexity. Measures introduced during that period, including movement restrictions, reduced travel, and changes in daily behavior, likely affected both disease transmission and routine surveillance systems [15]. In the case of VBDs, reduced mobility and fewer outdoor activities may have lowered opportunities for exposure in some settings, while pressure on health services may also have delayed diagnosis or led to underreporting of non-COVID conditions [7,16].

Much of the existing literature on the COVID-19 period has focused on its direct effects on respiratory and other viral infections. By comparison, less attention has been given to how the pandemic may have influenced the incidence, mortality, and spatiotemporal patterns of VBDs. Clarifying these indirect effects is important not only for interpreting recent epidemiological changes, but also for strengthening surveillance systems so they can remain functional during future public health emergencies [17,18].

This study aimed to provide a comprehensive analysis of the burden and epidemiological characteristics of vector-borne diseases in China from 2004 to 2023. Specifically, we sought to: (1) characterize long-term temporal trends in incidence and mortality; (2) describe seasonal, spatial, and age-specific patterns using the most granular data available; (3) compare reported incidence and mortality during the pre-COVID-19 period (2013–2019) and the COVID-19 era (2020–2023); and (4) evaluate departures from expected pre-COVID temporal patterns using interrupted time-series analysis and forecasting models. We hypothesized that the COVID-19 era would be associated with measurable changes in reported epidemiological patterns, although the magnitude and direction of these changes might vary across diseases and could not be attributed solely to COVID-19-related interventions.

## Materials and methods

### Ethical Statement

This study used de-identified surveillance data and publicly available aggregated reports. No individual-level identifiable information was accessed, and no direct contact with human participants occurred. Therefore, informed consent was not required, and the study was exempt from formal institutional review under applicable local regulations.

### Data source and study population

Epidemiological data used in this study were obtained from two complementary sources. Detailed province- and age-specific case and death records for vector-borne diseases (VBDs) between 2004 and 2020 were extracted from the National Infectious Disease Surveillance System (NIDSS), maintained by the Chinese Center for Disease Control and Prevention (China CDC). This dataset enabled comprehensive spatial, temporal, and demographic analyses during that period. For 2021–2023, national aggregate VBD statistics, including monthly and annual data, were obtained from official monthly bulletins and annual reports of the National Health Commission (NHC) of China. Therefore, spatial and age-specific analyses were restricted to 2004–2020, while national temporal analyses, interrupted time-series analyses, and SARIMA forecasting were extended through 2023. This mixed-data approach was adopted to leverage the most granular data available while maintaining continuity for long-term trend evaluation. All analyses were designed to clearly distinguish between data sources and timeframes to avoid bias or misinterpretation [19,20].

### Study period and data management

Different analyses used different observation windows according to data availability and analytical purpose. Annual national trend analyses used data from 2004–2023. Monthly interrupted time-series and forecasting analyses used national monthly data from 2013–2023, with 2013–2019 treated as the pre-COVID-19 baseline and 2020–2023 treated as the COVID-19 era. Province-level spatial and age-specific analyses were limited to 2004–2020 because only national aggregate data were available for 2021–2023.

The stratification allowed for assessing the pandemic's potential impacts on VBD trends and surveillance systems. Data were aggregated monthly and annually by disease, province, and age group, and managed using R software (version 4.4.3) [21].

### Temporal trends analysis

We analyzed annual incidence and mortality rates per 100,000 population using Joinpoint regression analysis to determine significant changes in disease trends. Joinpoint analysis provided the annual percent change (APC) and identified statistically significant breakpoints in disease incidence trends [22]. The analysis was performed using Joinpoint software (version 5.4.0, National Cancer Institute, USA) [23]. For consistent visual presentation across figures, the fitted trends and joinpoints identified from the Joinpoint analysis were exported and replotted.

### Seasonality analysis

Seasonal-trend decomposition based on Loess (STL) was employed to dissect monthly incidence data into seasonal, trend, and residual components. STL decomposition facilitated the identification of seasonal patterns and long-term changes [24]. This analysis was conducted in R software using the “forecast” and “stats” packages [25].

### Spatial distribution and clustering analysis

To show province-level variations in monthly and annual incidence and mortality, we generated spatiotemporal heatmaps. Spatial dependence was assessed using Global Moran’s I and Local Indicators of Spatial Association (LISA) in Python with standard spatial analysis libraries, which were used to identify significant local clusters and spatial outliers [26,27]. In the LISA maps, high–high areas were considered hotspots, low–low areas were considered coldspots, and high–low and low–high areas were treated as spatial outliers. Statistical significance was evaluated using permutation tests, with P < 0.05 taken to indicate significance. All spatial analyses were conducted programmatically using Python.

### Demographic analysis

Age-specific heatmaps were used to display differences in incidence patterns across age groups and study periods. These visualizations were produced in R using the ggplot2 package [28].

### Interrupted time-series analysis

To examine changes in monthly incidence and mortality during the COVID-19 period, we conducted interrupted time-series (ITS) analyses using segmented regression models, with January 2020 defined as the interruption point [29]. Each model included an underlying pre-intervention trend, a level-change term for the COVID-19 period, and a term representing the change in slope after the interruption. Month fixed effects were added to account for seasonality. Because monthly surveillance data may show heteroskedasticity and serial correlation, we used Newey–West standard errors with 12 lags to obtain robust estimates. Counterfactual trends were projected by extending the pre-COVID trajectory beyond January 2020 under a no-interruption assumption. As monthly mortality counts were low for several diseases, particularly in stratified analyses, mortality-related ITS results were interpreted with caution [30].

### Time-series forecasting analysis

To estimate expected monthly incidence and mortality trajectories during 2020–2023 based on pre-COVID patterns, we fitted seasonal autoregressive integrated moving average (SARIMA) models to monthly data from January 2015 to December 2019 for each disease and outcome [31]. The fitted models were then used to generate forecasts for January 2020 to December 2023, together with 95% prediction intervals. Observed values during 2020–2023 were compared with SARIMA-based expected trajectories to assess departures from pre-COVID-19 temporal patterns. Forecast performance metrics and selected model orders are provided in S4 Table. SARIMA modelling was implemented in R using standard time-series packages [32]. As a sensitivity analysis, we repeated the ITS models using monthly data from January 2015 to December 2023 while retaining the same interruption point and model specification, to assess whether the main ITS findings were sensitive to the length of the observation window used in the model.

### Statistical considerations

Key epidemiological metrics were incidence rate, mortality rate (per 100,000 population), and annual percent change (APC). Percentage changes between periods (2020–2023 vs. 2013–2019) were calculated with 95% confidence intervals (CIs). Statistical significance was assessed using two-proportion tests, and a two-sided P value < 0.05 was considered statistically significant. Statistical analyses were performed using R software (version 4.2.1) [21].

## Results

### Overall temporal trends of vector-borne diseases

Between 2004 and 2020, significant changes occurred in the temporal trends and disease composition of vector-borne diseases (VBDs) in China (Fig 1). Annual cases and deaths showed decreasing trends, particularly after 2010. The overall annual incidence rate showed a steady downward trend, with the sharpest declines seen in malaria and dengue fever. Mortality also decreased over the study period, suggesting a sustained reduction in disease burden. Joinpoint regression analysis further quantified these trends, revealing statistically significant annual percent changes (APC) across different disease categories (Fig 2; S1 Table). Notably, dengue fever and malaria showed distinct periods of rapid decline, especially in the later years of the study period.

**Fig 1:**
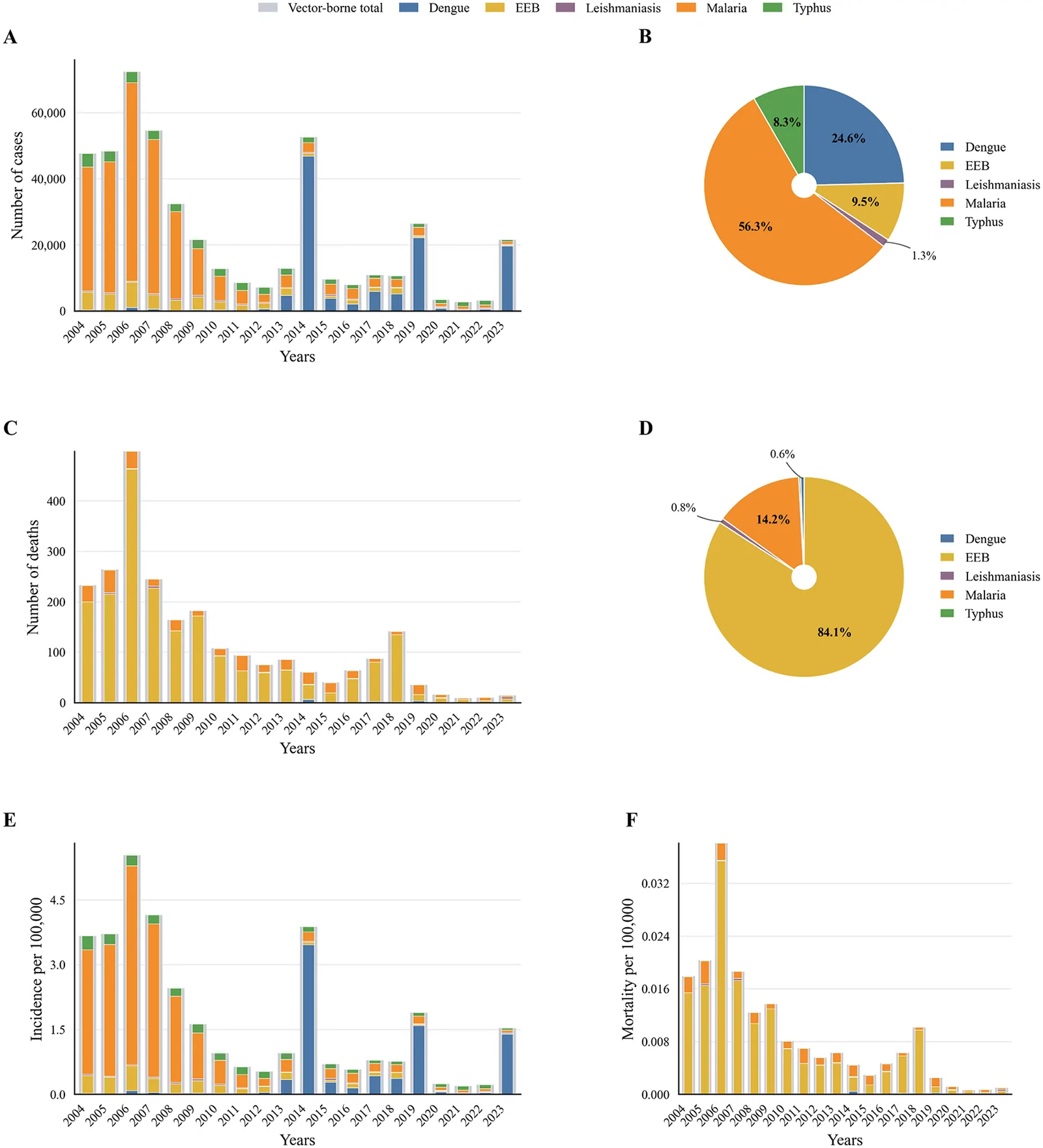
Temporal trends and composition of vector-borne diseases in China from 2004 to 2023. A shows Annual number of cases; B shows Proportion of total cases by disease type; C shows Annual number of deaths; D shows Proportion of total deaths by disease type; E shows Annual incidence per 100,000 population; F shows Annual mortality per 100,000 population.

**Fig 2:**
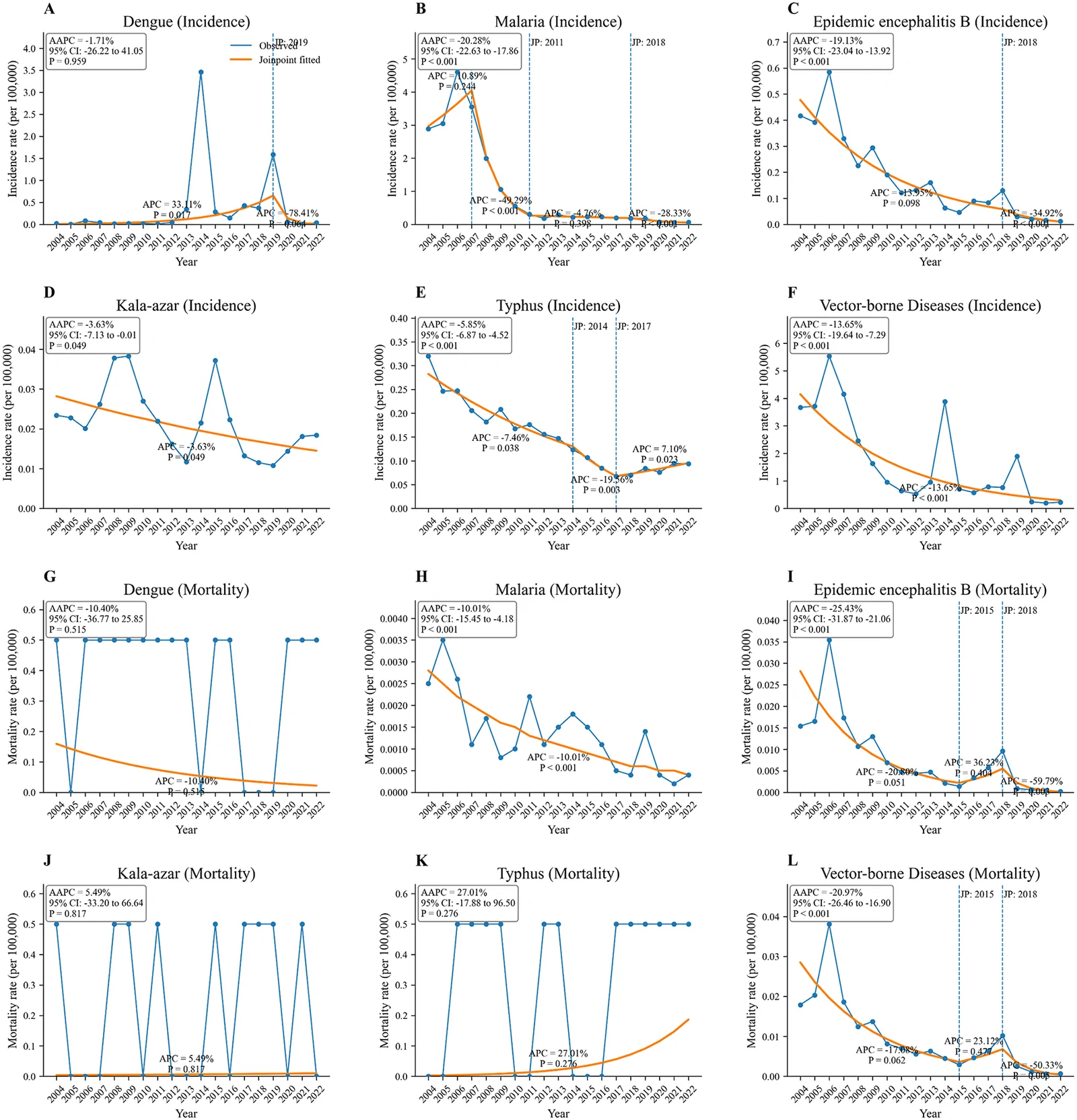
Joinpoint regression analysis of annual incidence rates of vector-borne diseases in China from 2004 to 2023. Panels A–F show incidence trends, and panels G–L show mortality trends for dengue, malaria, Japanese encephalitis, kala-azar, typhus, and overall vector-borne diseases. Observed annual rates are shown as blue dots and lines, while orange lines represent Joinpoint-fitted trends. Dashed vertical lines indicate detected joinpoints. APC and AAPC are presented in each panel.

### Seasonal and cyclic patterns

Seasonal-trend decomposition using LOESS (STL) showed clear seasonal variation in monthly reported VBD cases from 2004 to 2020, together with a general downward trend over time (Fig 3). Dengue fever and epidemic encephalitis B (EEB) showed marked peaks in summer and autumn, indicating strong seasonality, whereas diseases such as leishmaniasis and typhus had less obvious seasonal patterns.

**Fig 3:**
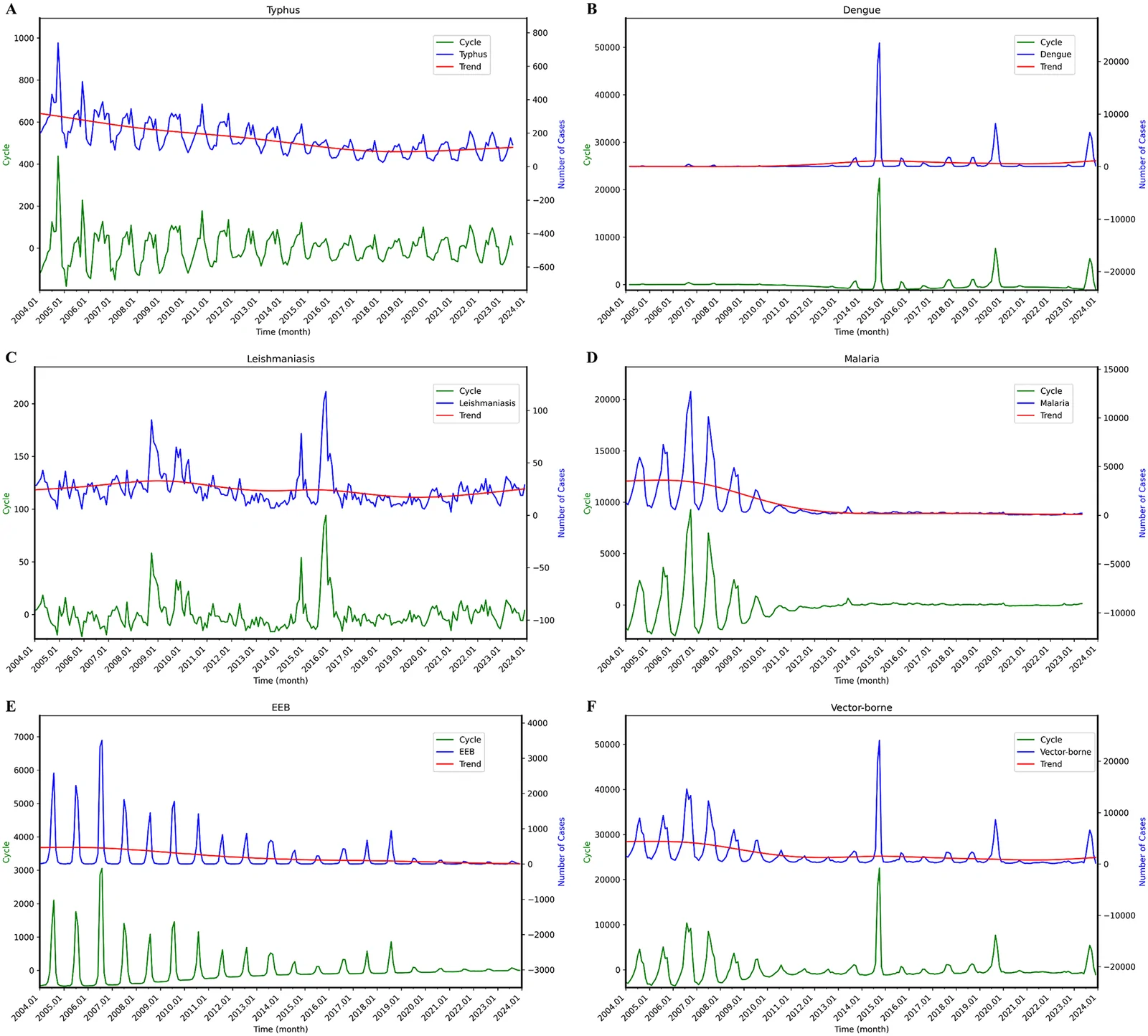
Seasonal-trend decomposition of monthly reported cases for major vector-borne diseases in China from 2004 to 2020. **(A)** Typhus; **(B)** Dengue; **(C)** Leishmaniasis; **(D)** Malaria; **(E)** Epidemic encephalitis B (EEB); **(F)** Total vector-borne diseases. Each panel shows the original time series (red), long-term trend (blue), and seasonal/cyclic component (green) based on monthly surveillance data.

### Spatial and age-specific distributions

Heatmaps of incidence and mortality from 2004 to 2020 showed clear differences across provinces, with consistently higher burdens in southern China, particularly for dengue fever, malaria, and EEB (Fig 4). Mortality patterns broadly mirrored incidence patterns, indicating persistent regional heterogeneity. Further spatial and demographic analyses (Fig 5) showed that dengue had concentrated clusters in southern provinces with age-specific peaks among young adults, malaria was more prominent in southwestern regions, EEB clustered mainly in central and eastern provinces, typhus had a broader distribution across central regions, and leishmaniasis showed smaller discrete clusters in western China. Global spatial autocorrelation analysis indicated significant positive clustering for leishmaniasis (Global Moran’s I = 0.251, P = 0.019), dengue (Global Moran’s I = 0.491, P < 0.001), and EEB (Global Moran’s I = 0.491, P < 0.001), whereas malaria (Global Moran’s I = 0.117, P = 0.219) and typhus (Global Moran’s I = 0.030, P = 0.619) did not show statistically significant global spatial autocorrelation. LISA maps further illustrated the local spatial patterns of hotspots, coldspots, and spatial outliers.

**Fig 4:**
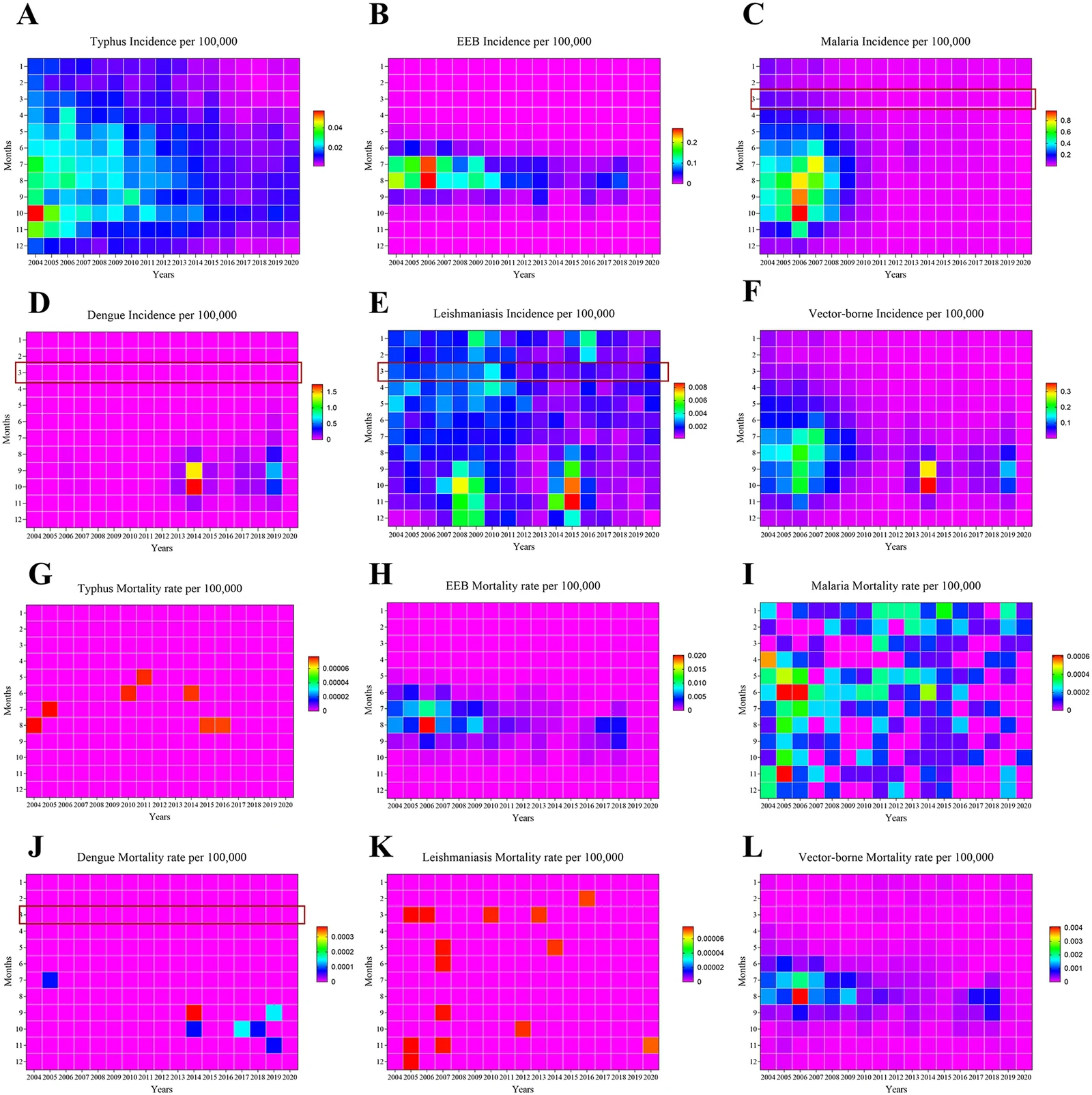
Monthly and yearly heatmap visualization of incidence and mortality rates for vector-borne diseases in China, 2004–2020. **(A–F)** Monthly incidence rates per 100,000 for typhus, EEB, malaria, dengue, leishmaniasis, and all vector-borne diseases; **(G–L)** Corresponding mortality rates per 100,000.

**Fig 5:**
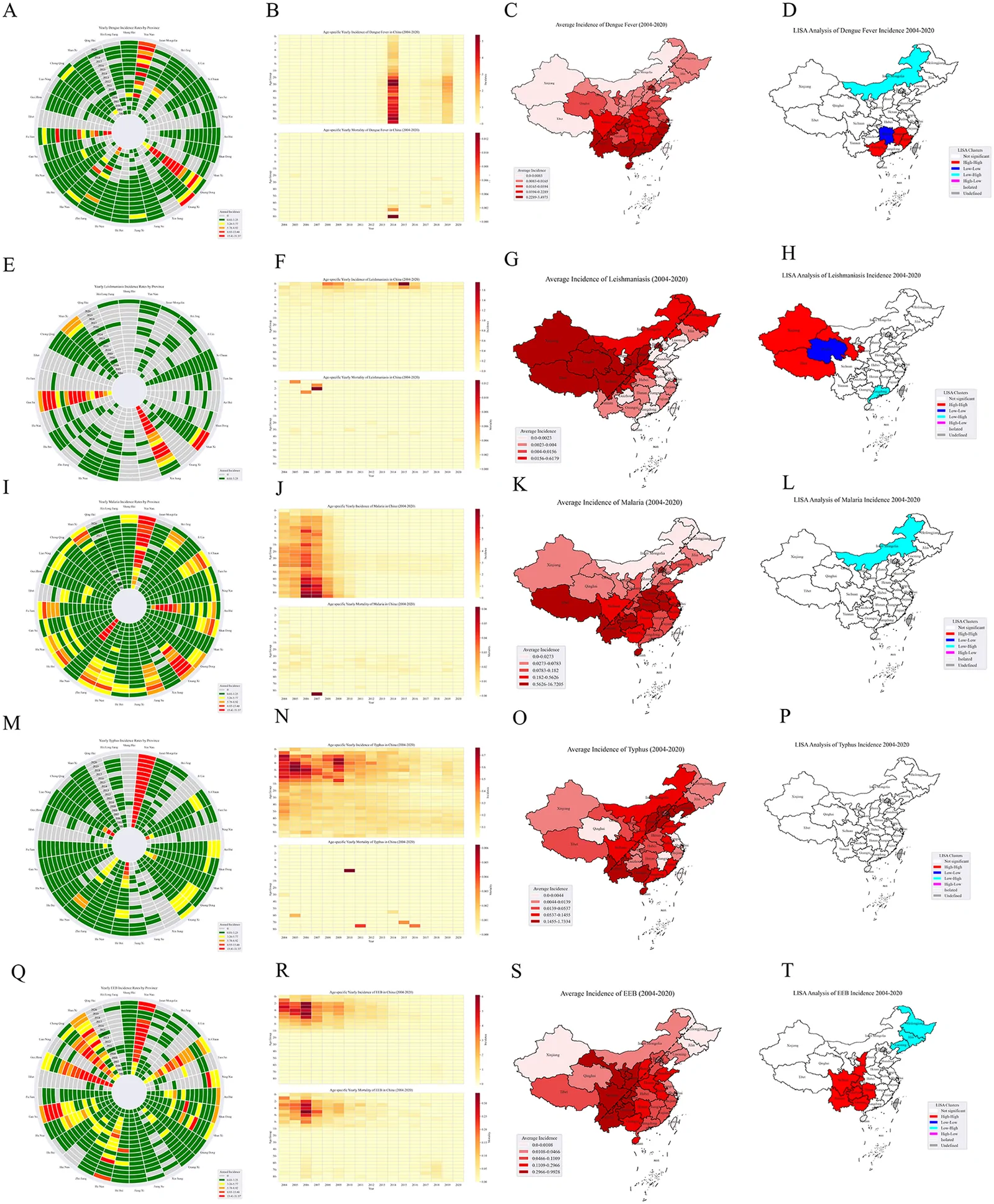
Multidimensional analysis of the incidence patterns of five major vector-borne diseases in China from 2004 to 2020. **(A–D)** Dengue fever; **(E–H)** Leishmaniasis; **(I–L)** Malaria; **(M–P)** Typhus; **(Q–T)** Epidemic encephalitis B (EEB). Each row includes: (A, E, I, M, Q) circular heatmaps showing annual incidence levels across provinces; (B, F, J, N, R) age-specific incidence heatmaps by year (y-axis: age groups, x-axis: years); (C, G, K, O, S) average incidence distribution across provinces (spatial maps); (D, H, L, P, **T)** Local Indicators of Spatial Association (LISA) cluster maps indicating spatial autocorrelation. High-high (HH) areas indicate hotspots, low-low (LL) areas indicate coldspots, and high-low (HL) and low-high (LH) areas indicate spatial outliers. Only areas with pseudo-P < 0.05 based on permutation tests are classified into LISA categories; all other areas are shown as non-significant. Base map source: Natural Earth, Admin 1 – States, Provinces vector dataset. Natural Earth map data are in the public domain. Source: https://www.naturalearthdata.com/downloads/10m-cultural-vectors/10m-admin-1-states-provinces/; Terms of use: https://www.naturalearthdata.com/about/terms-of-use/.

### Interrupted time-series analysis of incidence

Before conducting interrupted time-series analyses, we first compared the average annual incidence and mortality rates during the COVID-19 period with those in the pre-COVID period. As shown in Table 1, the average annual incidence of overall vector-borne diseases decreased from 1.3612 per 100,000 population in 2013–2019 to 0.5463 per 100,000 population in 2020–2023, corresponding to a 59.86% reduction. Marked decreases were also observed for epidemic encephalitis B, malaria, and dengue incidence, whereas leishmaniasis showed little change in average annual incidence. Mortality rates also declined for overall vector-borne diseases, epidemic encephalitis B, malaria, and dengue, although mortality estimates for several diseases were based on small numbers of deaths and should be interpreted cautiously. These descriptive comparisons provide an overall summary of changes between the two periods but do not account for pre-existing temporal trends or autocorrelation; therefore, interrupted time-series models were further used to assess immediate level changes and post-COVID slope changes after January 2020.

**Table 1:** Changes in the average yearly incidence and mortality rates of vector-borne diseases in 2020–2023 compared to 2013–2019 in China.

Diseases	Average yearly incidence		Average yearly cases (n)		Incidence		Average yearly mortality rates		Average yearly deaths (n)		Mortality	
	2013–2019	2020–2023	2013–2019	2020–2023	Percentage change^a^ (95% CI)	*P*-value^b^	2013–2019	2020–2023	2013–2019	2020–2023	Percentage change^c^ (95% CI)	*P*-value^b^
Typhus	0.0973	0.0764	1336.14	1077.00	-21.51 (-24.29 to -18.62)	<0.001	0.00003	0.00000	0.43	0	NA	NA
Dengue	0.9435	0.3724	12950.29	5250.25	-60.52 (-61.11 to -59.92)	<0.001	0.00012	0.00002	1.71	0.25	-85.80 (-98.15 to 9.22)	0.040
Leishmaniasis	0.0182	0.0184	250.57	259.75	0.95 (-6.51 to 9.00)	0.814	0.00003	0.00011	0.43	1.5	240.84 (-14.76 to 1262.85)	0.085
Malaria	0.2161	0.0627	2966.43	883.75	-70.99 (-72.00 to -69.94)	<0.001	0.00116	0.00032	15.86	4.5	-72.36 (-83.21 to -54.53)	<0.001
EEB	0.0859	0.0163	1180.00	230.25	-81.00 (-82.25 to -79.66)	<0.001	0.00399	0.00043	54.86	6	-89.35 (-92.95 to -83.91)	<0.001
Overall	1.3612	0.5463	18683.43	7701.00	-59.86 (-60.36 to -59.36)	<0.001	0.00534	0.00087	73.29	12.25	-83.72 (-87.86 to -78.18)	<0.001

CI, Confidence interval; EEB, epidemic encephalitis B.

aChanges = (x1 − x2)/x2 × 100%, where x1 is the average yearly incidence between 2020 and 2023, and x2 is the average yearly incidence in the previous 7 years (2013–2019.)

^b^The P-value was computed using two proportional tests.

cChanges = (x1 − x2)/x2 × 100%, where x1 is the average yearly mortality rates between 2020 and 2023, and x2 is the average yearly mortality rates in the previous 7 years (2013–2019).

Interrupted time-series analyses showed heterogeneous changes in monthly incidence across vector-borne diseases after January 2020 (Fig 6). The immediate level-change and post-interruption slope-change estimates for incidence and mortality across all diseases are summarized in Table 2. Complete ITS estimates, including the pre-interruption trends, are provided in S2 Table. In the main analysis, malaria incidence showed a significant immediate decline at the onset of the COVID-19 period, and overall vector-borne disease incidence also showed a significant immediate level decrease. Dengue incidence showed a downward level change, although the statistical evidence was weaker in the main model. Positive post-COVID slope changes were observed for typhus and leishmaniasis incidence, suggesting gradual rebounds relative to pre-COVID trajectories. In contrast, no significant immediate level or slope change was identified for epidemic encephalitis B incidence. For mortality, monthly series were sparse for several diseases and contained multiple zero values; therefore, these estimates should be interpreted cautiously. Most mortality ITS coefficients were not statistically robust, although some disease-specific changes were observed in individual models. The sensitivity ITS analysis restricted to 2015–2023 showed patterns broadly consistent with the main analysis (S1 Fig; S3 Table). In particular, immediate declines in malaria incidence and total vector-borne disease incidence remained evident, dengue incidence showed a clearer immediate decrease, typhus and leishmaniasis continued to show positive post-COVID slope changes, and epidemic encephalitis B remained largely unchanged. Mortality estimates remained less stable because of sparse monthly deaths.

**Table 2:** Summary of interrupted time-series estimates for monthly incidence and mortality rates of vector-borne diseases in China, 2013–2023.

Disease	Incidence				Mortality(×10^6^)			
	Immediate level change (95% CI)	*P*	Post-interruption slope change (95% CI)	*P*	Immediate level change (95% CI)	*P*	Post-interruption slope change (95% CI)	*P*
Typhus	0.00150 (−0.000292 to 0.00330)	0.100	0.000145 (0.000099 to 0.000192)	<0.001	0.00 (−3.00 to 3.00)	0.846	0.00^a^ (0.00^a^ to 0.00^a^)	0.243
Dengue	−0.0828 (−0.1741 to 0.0085)	0.076	0.00270 (−0.00110 to 0.00650)	0.170	−11.00 (−23.00 to 0.00^a^)	0.048	0.00^a^ (0.00^a^ to 0.00^a^)	0.918
Leishmaniasis	0.000155 (−0.000521 to 0.000831)	0.653	0.0000220 (0.0000040 to 0.0000410)	0.020	−2.00 (−11.00 to 7.00)	0.668	1.00 (0.00^a^ to 1.00)	<0.001
Malaria	−0.00930 (−0.0127 to −0.00590)	<0.001	0.000191 (−0.000005 to 0.000387)	0.056	−33.00 (−82.00 to 15.00)	0.177	2.00 (0.00 to 3.00)	0.063
EEB	−0.00360 (−0.00970 to 0.00250)	0.249	0.000058 (−0.000096 to 0.000212)	0.458	−398.00 (−893.00 to 97.00)	0.115	−4.00 (−14.0 to 6.00)	0.451
Overall	−0.0893 (−0.1772 to −0.0014)	0.046	0.00280 (−0.000905 to 0.00650)	0.139	−376.00 (−776.00 to 23.00)	0.065	−3.00 (−10.0 to 4.00)	0.446

ITS, interrupted time series; CI, confidence interval; EEB, epidemic encephalitis B.

Note: Estimates were obtained from segmented regression models with January 2020 as the interruption point. Models included a pre-interruption trend, month fixed effects, and Newey–West standard errors with 12 lags. Mortality coefficients and 95% CIs were multiplied by 10⁶ for readability. ^a^ Values marked with superscript a were rounded to 0.00 in the exported model output and should not be interpreted as exact zeros. Mortality estimates should be interpreted cautiously because several disease-specific series contained sparse monthly deaths and multiple zero values.

**Fig 6:**
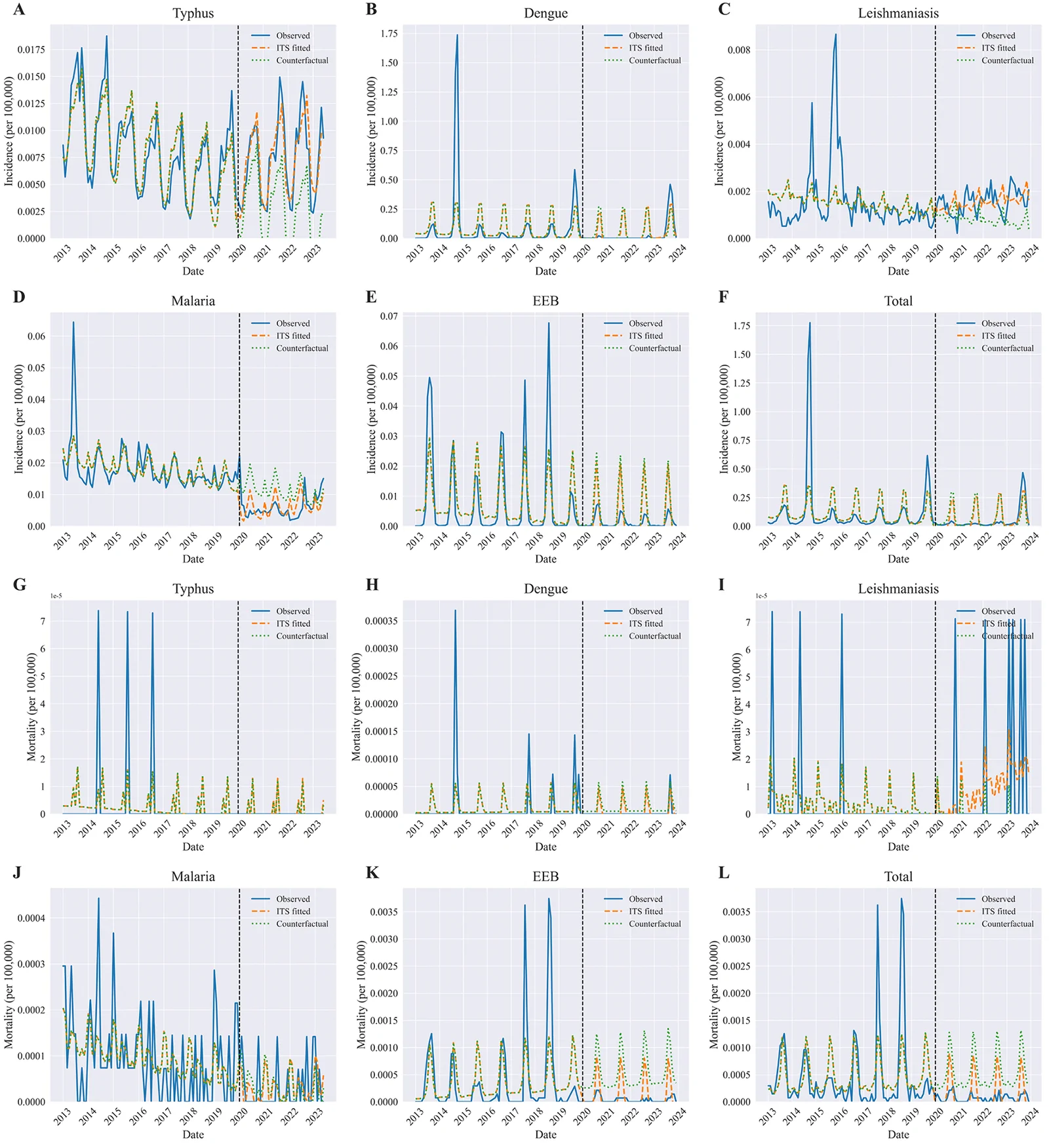
Interrupted time-series analysis of monthly incidence and mortality rates of vector-borne diseases in China, 2013–2023. **(A–F)** Monthly incidence rates for typhus, dengue, leishmaniasis, malaria, epidemic encephalitis B (EEB), and all vector-borne diseases; (G–L) corresponding monthly mortality rates. Blue solid lines represent observed values, orange dashed lines indicate fitted values from the interrupted time-series models, and green dotted lines represent counterfactual trends projected from the pre-COVID period. Vertical dashed lines indicate the onset of the COVID-19 period in January 2020.

### SARIMA-based forecasting analysis

Fig 7 shows the observed monthly incidence and mortality rates during 2020–2023 together with SARIMA-predicted trajectories derived from pre-COVID-19 data (2015–2019). For incidence, observed values for dengue, malaria, epidemic encephalitis B, and total vector-borne diseases were generally below SARIMA-based expected trajectories during substantial parts of 2020–2023, whereas leishmaniasis showed persistently higher observed incidence than predicted in much of the post-2020 period. Typhus showed alternating periods above and below the SARIMA-based expected trajectory.

**Fig 7:**
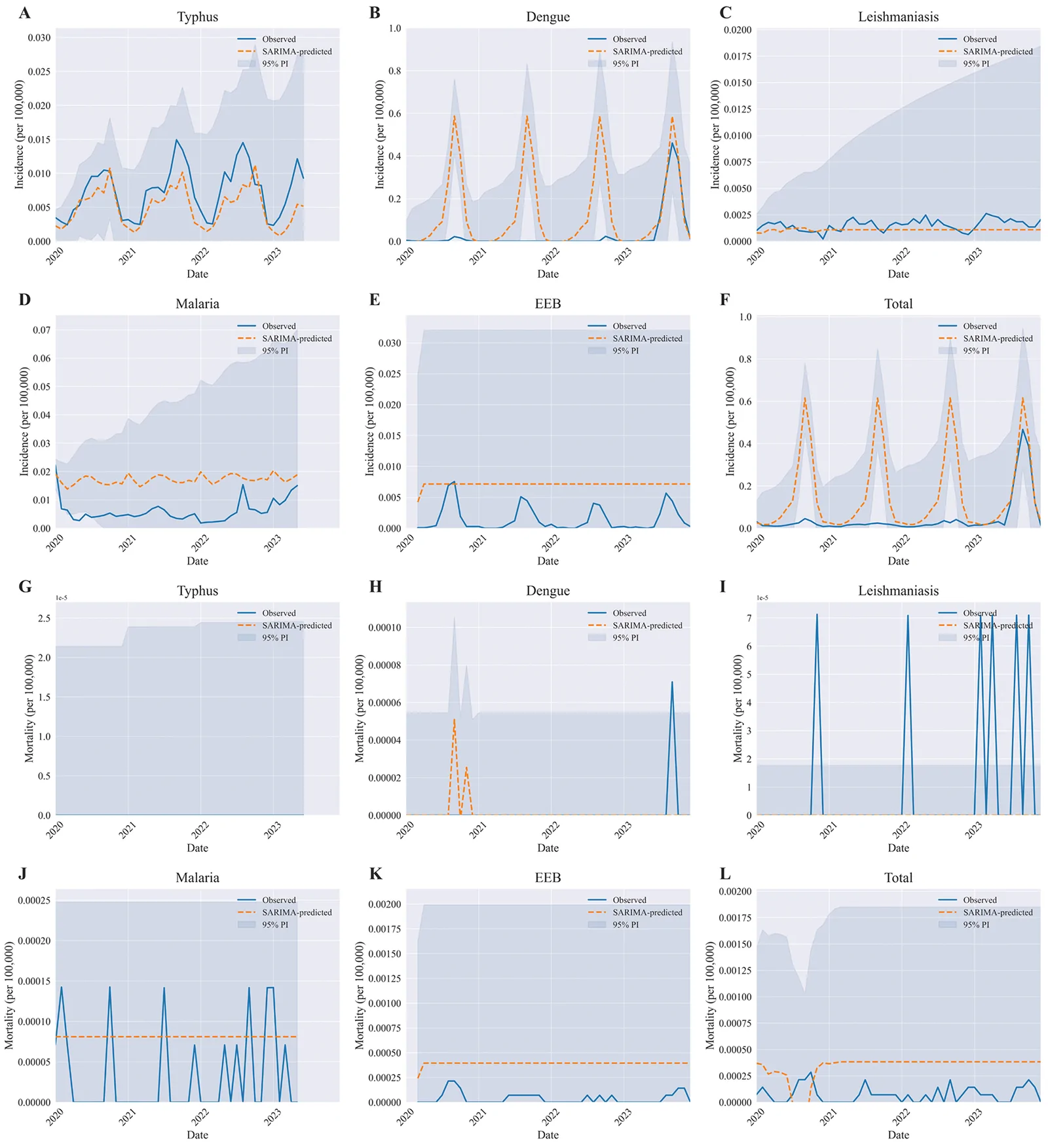
Observed and SARIMA-predicted monthly incidence and mortality rates of vector-borne diseases in China, 2020–2023. **(A–F)** Monthly incidence rates for typhus, dengue, leishmaniasis, malaria, epidemic encephalitis B (EEB), and all vector-borne diseases; (G–L) corresponding monthly mortality rates. Orange dashed lines represent SARIMA-predicted mean values, blue solid lines indicate observed values, and shaded areas represent the 95% prediction intervals. Predictions were generated using seasonal autoregressive integrated moving average (SARIMA) models trained on monthly baseline data from 2015 to 2019.

For mortality, the monthly series were sparse for several diseases and included many zero values; therefore, disease-specific deviations from SARIMA-predicted values should be interpreted cautiously. Overall, these differences are interpreted as departures from expected pre-pandemic temporal patterns.

## Discussion

This study provides a comprehensive analysis of reported vector-borne diseases (VBDs) in China from 2004 to 2023. Overall, incidence and mortality declined substantially over the study period, particularly for malaria, dengue, and epidemic encephalitis B (EEB), and these declines were also reflected in the Joinpoint analyses. During 2020–2023, reported incidence and mortality were markedly lower than during 2013–2019. However, these reductions should be interpreted cautiously and should not be attributed solely to COVID-19-related interventions. Previous studies have reported associations between COVID-19-related public health measures, changes in mobility, and changes in the reported incidence of infectious diseases. These findings provide plausible contextual explanations for the patterns observed in our analysis. However, our ecological study did not directly measure intervention exposure, individual behavior, healthcare access, or reporting completeness, and therefore could not determine the causes of the observed reductions or quantify the contribution of any specific factor [33,34].

The observed reductions in reported VBDs coincided with major behavioral and public health changes during the COVID-19 era, including reduced mobility, travel restrictions, and altered social contact patterns, all of which may have affected vector exposure and transmission opportunities. At the same time, several diseases—particularly malaria and EEB—had already shown substantial downward trends before 2020, reflecting long-term control efforts and, in the case of malaria, the broader elimination process in China. Therefore, the lower reported burden observed during 2020–2023 cannot be interpreted as a simple or direct effect of COVID-19 control measures alone [16,35].

Our findings are broadly consistent with reports from other countries showing reduced reported incidence of several infectious diseases during the COVID-19 era [36]. However, the magnitude and interpretation of these changes are likely context-specific. In addition, although leishmaniasis incidence remained relatively stable, mortality showed a non-significant upward tendency during 2020–2023. This finding should not be overinterpreted, but it suggests that continued attention to severe outcomes and case management remains warranted in endemic settings [37].

Our study has several limitations. First, although we used nationwide surveillance data, underreporting, delayed diagnosis, and changes in healthcare-seeking behavior during the COVID-19 era may have affected reported incidence and mortality, leading to possible surveillance bias. Diversion of diagnostic resources and reduced healthcare utilization during this period may also have contributed to lower reported case counts independent of changes in true transmission. Second, the present study was not designed to quantitatively disentangle the relative contributions of COVID-19-related interventions, pre-existing long-term trends, disease-specific control programs, and underreporting. Therefore, the observed reductions should be interpreted as changes in reported epidemiological patterns rather than definitive estimates of changes in true transmission [34]. Third, spatial and age-specific analyses could only be conducted through 2020 because more granular province-level and age-stratified data were not available for 2021–2023. Finally, the SARIMA-based forecasting analysis was intended to characterize departures from expected pre-pandemic temporal patterns rather than to provide a fully validated real-time outbreak prediction framework [38].

Future studies should integrate environmental, climatic, and socioeconomic factors into VBD prediction models to improve forecast performance, especially under changing ecological conditions. More detailed data on age-specific mortality, healthcare utilization, and reporting completeness would also help clarify the mechanisms underlying reported changes during large-scale public health emergencies.

## Supporting information

S1 Table Joinpoint regression analysis of annual incidence and mortality trends for vector-borne diseases in China, 2004–2023. (DOCX)

S2 Table Interrupted time-series estimates for monthly incidence and mortality rates of vector-borne diseases in China, 2013–2023. (DOCX)

S3 Table Interrupted time-series estimates for monthly incidence and mortality rates of vector-borne diseases in China, 2015–2023. (DOCX)

S4 Table Forecast performance metrics and selected SARIMA model orders for monthly incidence and mortality series of vector-borne diseases in China. (DOCX)

S1 Fig Interrupted time-series analysis of monthly incidence and mortality rates of vector-borne diseases in China, 2015–2023. (A–F) Monthly incidence rates for typhus, dengue, leishmaniasis, malaria, epidemic encephalitis B (EEB), and all vector-borne diseases; (G–L) corresponding monthly mortality rates. Blue solid lines represent observed values, orange dashed lines indicate fitted values from the interrupted time-series models, and green dotted lines represent counterfactual trends projected from the pre-COVID period. Vertical dashed lines indicate the onset of the COVID-19 period in January 2020.
(PNG)
