# Supplementary material for: Temporal trends in reported vector-borne diseases in China before and after the onset of COVID-19 from 2004 to 2023
Source: PLoS Negl Trop Dis. 2026 Oct 21;20(10):e0014745. doi: 10.1371/journal.pntd.0014745 (PMC13750651; doi:10.1371/journal.pntd.0014745)
Supplement: Supplementary file 1 [file pntd.0014745.s001.docx]

**S1 Table.** Joinpoint regression analysis of annual incidence and mortality trends for vector-borne diseases in China, 2004–2023.

| Disease | Outcome | Period | Trend measure | Estimate (%) | 95% CI | *P* |
| --- | --- | --- | --- | --- | --- | --- |
| Dengue | Incidence | 2004–2019 | APC | 33.11 | 12.74 to 551.14 | 0.017 |
| Dengue | Incidence | 2019–2022 | APC | -78.41 | -99.13 to 3.33 | 0.064 |
| Dengue | Incidence | 2004–2022 | AAPC | -1.71 | -26.22 to 41.05 | 0.959 |
| Malaria | Incidence | 2004–2007 | APC | 10.89 | -10.16 to 43.89 | 0.244 |
| Malaria | Incidence | 2007–2011 | APC | -49.29 | -59.61 to -39.89 | <0.001 |
| Malaria | Incidence | 2011–2018 | APC | -4.76 | -10.15 to 19.09 | 0.398 |
| Malaria | Incidence | 2018–2022 | APC | -28.33 | -46.19 to -18.79 | <0.001 |
| Malaria | Incidence | 2004–2022 | AAPC | -20.28 | -22.63 to -17.86 | <0.001 |
| Epidemic encephalitis B | Incidence | 2004–2018 | APC | -13.95 | -17.48 to 19.67 | 0.098 |
| Epidemic encephalitis B | Incidence | 2018–2022 | APC | -34.92 | -62.45 to -18.13 | <0.001 |
| Epidemic encephalitis B | Incidence | 2004–2022 | AAPC | -19.13 | -23.04 to -13.92 | <0.001 |
| Kala-azar | Incidence | 2004–2022 | APC | -3.63 | -7.13 to -0.01 | 0.049 |
| Kala-azar | Incidence | 2004–2022 | AAPC | -3.63 | -7.13 to -0.01 | 0.049 |
| Typhus | Incidence | 2004–2014 | APC | -7.46 | -8.86 to -2.31 | 0.038 |
| Typhus | Incidence | 2014–2017 | APC | -19.56 | -24.74 to -10.54 | 0.003 |
| Typhus | Incidence | 2017–2022 | APC | 7.1 | 1.29 to 25.11 | 0.023 |
| Typhus | Incidence | 2004–2022 | AAPC | -5.85 | -6.87 to -4.52 | <0.001 |
| Vector-borne Diseases | Incidence | 2004–2022 | APC | -13.65 | -19.64 to -7.29 | <0.001 |
| Vector-borne Diseases | Incidence | 2004–2022 | AAPC | -13.65 | -19.64 to -7.29 | <0.001 |
| Dengue | Mortality | 2004–2022 | APC | -10.4 | -36.77 to 25.85 | 0.515 |
| Dengue | Mortality | 2004–2022 | AAPC | -10.4 | -36.77 to 25.85 | 0.515 |
| Malaria | Mortality | 2004–2022 | APC | -10.01 | -15.45 to -4.18 | <0.001 |
| Malaria | Mortality | 2004–2022 | AAPC | -10.01 | -15.45 to -4.18 | <0.001 |
| Epidemic encephalitis B | Mortality | 2004–2015 | APC | -20.8 | -51.48 to 2.83 | 0.051 |
| Epidemic encephalitis B | Mortality | 2015–2018 | APC | 36.23 | -14.97 to 78.91 | 0.404 |
| Epidemic encephalitis B | Mortality | 2018–2022 | APC | -59.79 | -80.99 to -45.34 | 0.003 |
| Epidemic encephalitis B | Mortality | 2004–2022 | AAPC | -25.43 | -31.87 to -21.06 | <0.001 |
| Kala-azar | Mortality | 2004–2022 | APC | 5.49 | -33.20 to 66.64 | 0.817 |
| Kala-azar | Mortality | 2004–2022 | AAPC | 5.49 | -33.20 to 66.64 | 0.817 |
| Typhus | Mortality | 2004–2022 | APC | 27.01 | -17.88 to 96.50 | 0.276 |
| Typhus | Mortality | 2004–2022 | AAPC | 27.01 | -17.88 to 96.50 | 0.276 |
| Vector-borne Diseases | Mortality | 2004–2015 | APC | -17.08 | -43.17 to 29.65 | 0.062 |
| Vector-borne Diseases | Mortality | 2015–2018 | APC | 23.12 | -13.13 to 52.07 | 0.477 |
| Vector-borne Diseases | Mortality | 2018–2022 | APC | -50.33 | -73.25 to -36.62 | 0.005 |
| Vector-borne Diseases | Mortality | 2004–2022 | AAPC | -20.97 | -26.46 to -16.90 | <0.001 |
