# Supplementary material for: Temporal trends in reported vector-borne diseases in China before and after the onset of COVID-19 from 2004 to 2023
Source: PLoS Negl Trop Dis. 2026 Oct 21;20(10):e0014745. doi: 10.1371/journal.pntd.0014745 (PMC13750651; doi:10.1371/journal.pntd.0014745)
Supplement: Supplementary file 2 [file pntd.0014745.s002.docx]

**S2 Table.** Interrupted time-series estimates for monthly incidence and mortality rates of vector-borne diseases in China, 2013–2023.

| **Disease** | **Outcome** | **ITS parameter** | **Coefficient** | **95% CI** | ***P*** |
| --- | --- | --- | --- | --- | --- |
| Typhus | Incidence | Pre-COVID trend | -8.3e-05 | -0.000115 to -0.000050 | <0.001 |
| Typhus | Incidence | Immediate level change | 0.0015 | -0.000292 to 0.0033 | 0.100 |
| Typhus | Incidence | Post-COVID slope change | 0.000145 | 0.000099 to 0.000192 | <0.001 |
| Typhus | Mortality | Pre-COVID trend | -0.0 | -0.000000 to 0.000000 | 0.182 |
| Typhus | Mortality | Immediate level change | -0.0 | -0.000003 to 0.000003 | 0.846 |
| Typhus | Mortality | Post-COVID slope change | 0.0 | -0.000000 to 0.000000 | 0.243 |
| Dengue | Incidence | Pre-COVID trend | -0.000464 | -0.0031 to 0.0022 | 0.733 |
| Dengue | Incidence | Immediate level change | -0.0828 | -0.1741 to 0.0085 | 0.076 |
| Dengue | Incidence | Post-COVID slope change | 0.0027 | -0.0011 to 0.0065 | 0.170 |
| Dengue | Mortality | Pre-COVID trend | 0.0 | -0.000000 to 0.000000 | 0.873 |
| Dengue | Mortality | Immediate level change | -1.1e-05 | -0.000023 to -0.000000 | 0.048 |
| Dengue | Mortality | Post-COVID slope change | 0.0 | -0.000000 to 0.000000 | 0.918 |
| Leishmaniasis | Incidence | Pre-COVID trend | -1e-05 | -0.000030 to 0.000009 | 0.280 |
| Leishmaniasis | Incidence | Immediate level change | 0.000155 | -0.000521 to 0.000831 | 0.653 |
| Leishmaniasis | Incidence | Post-COVID slope change | 2.2e-05 | 0.000004 to 0.000041 | 0.020 |
| Leishmaniasis | Mortality | Pre-COVID trend | -0.0 | -0.000000 to -0.000000 | 0.003 |
| Leishmaniasis | Mortality | Immediate level change | -2e-06 | -0.000011 to 0.000007 | 0.668 |
| Leishmaniasis | Mortality | Post-COVID slope change | 1e-06 | 0.000000 to 0.000001 | <0.001 |
| Malaria | Incidence | Pre-COVID trend | -0.000106 | -0.000167 to -0.000046 | <0.001 |
| Malaria | Incidence | Immediate level change | -0.0093 | -0.0127 to -0.0059 | <0.001 |
| Malaria | Incidence | Post-COVID slope change | 0.000191 | -0.000005 to 0.000387 | 0.056 |
| Malaria | Mortality | Pre-COVID trend | -1e-06 | -0.000002 to -0.000000 | 0.023 |
| Malaria | Mortality | Immediate level change | -3.3e-05 | -0.000082 to 0.000015 | 0.177 |
| Malaria | Mortality | Post-COVID slope change | 2e-06 | -0.000000 to 0.000003 | 0.063 |
| EEB | Incidence | Pre-COVID trend | -7.1e-05 | -0.000214 to 0.000072 | 0.332 |
| EEB | Incidence | Immediate level change | -0.0036 | -0.0097 to 0.0025 | 0.249 |
| EEB | Incidence | Post-COVID slope change | 5.8e-05 | -0.000096 to 0.000212 | 0.458 |
| EEB | Mortality | Pre-COVID trend | 3e-06 | -0.000007 to 0.000012 | 0.581 |
| EEB | Mortality | Immediate level change | -0.000398 | -0.000893 to 0.000097 | 0.115 |
| EEB | Mortality | Post-COVID slope change | -4e-06 | -0.000014 to 0.000006 | 0.451 |
| Total | Incidence | Pre-COVID trend | -0.000713 | -0.0033 to 0.0019 | 0.589 |
| Total | Incidence | Immediate level change | -0.0893 | -0.1772 to -0.0014 | 0.046 |
| Total | Incidence | Post-COVID slope change | 0.0028 | -0.000905 to 0.0065 | 0.139 |
| Total | Mortality | Pre-COVID trend | 1e-06 | -0.000005 to 0.000007 | 0.740 |
| Total | Mortality | Immediate level change | -0.000376 | -0.000776 to 0.000023 | 0.065 |
| Total | Mortality | Post-COVID slope change | -3e-06 | -0.000010 to 0.000004 | 0.446 |

**Abbreviations:** ITS, interrupted time series; CI, confidence interval; EEB, epidemic encephalitis B. **Note:** Coefficient estimates were derived from segmented regression models including baseline temporal trend, immediate level change at January 2020, post-COVID slope change, and month fixed effects. Heteroskedasticity- and autocorrelation-consistent standard errors were estimated using a Newey–West type covariance estimator with 12 lags. Results for mortality outcomes should be interpreted cautiously for diseases with sparse monthly deaths and multiple zero values.
