# Supplementary material for: Temporal trends in reported vector-borne diseases in China before and after the onset of COVID-19 from 2004 to 2023
Source: PLoS Negl Trop Dis. 2026 Oct 21;20(10):e0014745. doi: 10.1371/journal.pntd.0014745 (PMC13750651; doi:10.1371/journal.pntd.0014745)
Supplement: Supplementary file 3 [file pntd.0014745.s003.docx]

**S3 Table.** Interrupted time-series estimates for monthly incidence and mortality rates of vector-borne diseases in China, 2015–2023.

| **Disease** | **Outcome** | **ITS parameter** | **Coefficient** | **95% CI** | ***P*** |
| --- | --- | --- | --- | --- | --- |
| Typhus | Incidence | Pre-COVID trend | -0.000044 | -0.000090 to 0.000002 | 0.063 |
| Typhus | Incidence | Immediate level change | 0.000563 | -0.0011 to 0.0022 | 0.505 |
| Typhus | Incidence | Post-COVID slope change | 0.000107 | 0.000052 to 0.000162 | <0.001 |
| Typhus | Mortality | Pre-COVID trend | 0 | -0.000000 to -0.000000 | <0.001 |
| Typhus | Mortality | Immediate level change | 0.000002 | -0.000000 to 0.000004 | 0.106 |
| Typhus | Mortality | Post-COVID slope change | 0 | 0.000000 to 0.000000 | 0.004 |
| Dengue | Incidence | Pre-COVID trend | 0.002 | 0.000106 to 0.0040 | 0.039 |
| Dengue | Incidence | Immediate level change | -0.1416 | -0.2131 to -0.0700 | <0.001 |
| Dengue | Incidence | Post-COVID slope change | 0.000655 | -0.0032 to 0.0045 | 0.739 |
| Dengue | Mortality | Pre-COVID trend | 0 | 0.000000 to 0.000001 | 0.005 |
| Dengue | Mortality | Immediate level change | -0.000019 | -0.000029 to -0.000010 | <0.001 |
| Dengue | Mortality | Post-COVID slope change | 0 | -0.000001 to 0.000000 | 0.128 |
| Leishmaniasis | Incidence | Pre-COVID trend | -0.000041 | -0.000074 to -0.000009 | 0.012 |
| Leishmaniasis | Incidence | Immediate level change | 0.000877 | 0.000098 to 0.0017 | 0.027 |
| Leishmaniasis | Incidence | Post-COVID slope change | 0.000053 | 0.000023 to 0.000084 | <0.001 |
| Leishmaniasis | Mortality | Pre-COVID trend | 0 | -0.000000 to 0.000000 | 0.420 |
| Leishmaniasis | Mortality | Immediate level change | -0.000003 | -0.000012 to 0.000006 | 0.552 |
| Leishmaniasis | Mortality | Post-COVID slope change | 0.000001 | 0.000000 to 0.000001 | 0.001 |
| Malaria | Incidence | Pre-COVID trend | -0.000102 | -0.000146 to -0.000058 | <0.001 |
| Malaria | Incidence | Immediate level change | -0.0094 | -0.0121 to -0.0067 | <0.001 |
| Malaria | Incidence | Post-COVID slope change | 0.000178 | -0.000001 to 0.000357 | 0.052 |
| Malaria | Mortality | Pre-COVID trend | -0.000001 | -0.000002 to 0.000001 | 0.299 |
| Malaria | Mortality | Immediate level change | -0.000038 | -0.000086 to 0.000011 | 0.125 |
| Malaria | Mortality | Post-COVID slope change | 0.000001 | -0.000001 to 0.000003 | 0.290 |
| EEB | Incidence | Pre-COVID trend | 0.000002 | -0.000190 to 0.000194 | 0.984 |
| EEB | Incidence | Immediate level change | -0.0053 | -0.0125 to 0.0018 | 0.142 |
| EEB | Incidence | Post-COVID slope change | -0.000013 | -0.000207 to 0.000182 | 0.899 |
| EEB | Mortality | Pre-COVID trend | 0.000005 | -0.000011 to 0.000021 | 0.559 |
| EEB | Mortality | Immediate level change | -0.000448 | -0.0011 to 0.000172 | 0.157 |
| EEB | Mortality | Post-COVID slope change | -0.000006 | -0.000022 to 0.000010 | 0.464 |
| Total | Incidence | Pre-COVID trend | 0.0019 | -0.000001 to 0.0037 | 0.050 |
| Total | Incidence | Immediate level change | -0.1505 | -0.2176 to -0.0835 | <0.001 |
| Total | Incidence | Post-COVID slope change | 0.000691 | -0.0030 to 0.0044 | 0.713 |
| Total | Mortality | Pre-COVID trend | 0.000004 | -0.000007 to 0.000015 | 0.494 |
| Total | Mortality | Immediate level change | -0.000445 | -0.000956 to 0.000067 | 0.089 |
| Total | Mortality | Post-COVID slope change | -0.000006 | -0.000017 to 0.000006 | 0.326 |

**Abbreviations:** ITS, interrupted time series; CI, confidence interval; EEB, epidemic encephalitis B. **Note:** This sensitivity analysis used monthly data from January 2015 to December 2023. Coefficients were derived from segmented regression models including baseline temporal trend, immediate level change at January 2020, post-COVID slope change, and month fixed effects. Heteroskedasticity- and autocorrelation-consistent standard errors were estimated using a Newey–West type covariance estimator with 12 lags. Mortality results for diseases with sparse monthly deaths should be interpreted cautiously.
