# Supplementary material for: Temporal trends in reported vector-borne diseases in China before and after the onset of COVID-19 from 2004 to 2023
Source: PLoS Negl Trop Dis. 2026 Oct 21;20(10):e0014745. doi: 10.1371/journal.pntd.0014745 (PMC13750651; doi:10.1371/journal.pntd.0014745)
Supplement: Supplementary file 4 [file pntd.0014745.s004.docx]

**S4 Table.** Forecast performance metrics and selected SARIMA model orders for monthly incidence and mortality series of vector-borne diseases in China

| **Disease** | **Outcome** | **RMSE** | **MAE** | **MAPE (%)** | **SARIMA order** | **Seasonal order** |
| --- | --- | --- | --- | --- | --- | --- |
| Typhus | Incidence | 0.0032 | 0.0026 | 34.08 | (0,1,0) | (2,1,0,12) |
| Typhus | Mortality | 2.6E-07 | 2.5E-07 | NA | (0,0,0) | (1,0,0,12) |
| Dengue | Incidence | 0.1922 | 0.1031 | 27613.54 | (1,0,1) | (0,1,0,12) |
| Dengue | Mortality | 1.31E-05 | 3.12E-06 | 100 | (0,0,0) | (0,0,1,12) |
| Leishmaniasis | Incidence | 0.0007 | 0.0006 | 41.33 | (0,1,0) | (0,0,1,12) |
| Leishmaniasis | Mortality | 2.51E-05 | 8.87E-06 | 100 | (0,0,0) | (0,0,0,12) |
| Malaria | Incidence | 0.0117 | 0.0112 | 276.75 | (0,1,0) | (1,0,1,12) |
| Malaria | Mortality | 7.21E-05 | 6.77E-05 | 28.54 | (0,0,0) | (0,0,0,12) |
| EEB | Incidence | 0.006 | 0.0057 | 2561.27 | (0,0,1) | (0,0,0,12) |
| EEB | Mortality | 0.000358 | 0.000353 | 363.52 | (0,0,1) | (0,0,0,12) |
| Total | Incidence | 0.1974 | 0.1111 | 454.63 | (1,0,1) | (0,1,0,12) |
| Total | Mortality | 0.000316 | 0.000305 | 286.32 | (0,0,2) | (0,0,1,12) |
